# Psychological distress and health perception in patients with a previous myocardial infarction or stroke: a national cross-sectional study

**DOI:** 10.1186/s12872-023-03422-5

**Published:** 2023-08-30

**Authors:** Aparna Narendrula, Kiran Ajani, Jacob Lang, Ellen Brinza, Chris T Longenecker

**Affiliations:** 1 New York University Grossman School of Medicine, 550 First Avenue, NBV 16 North 30, 10016 New York, NY USA; 2 The Ohio State University Wexner Medical Center, Columbus, OH USA; 3 New York–Presbyterian Hospital - Weill Cornell Medicine, New York, NY USA; 4 University of Colorado Anschutz Medical Center, Aurora, CO USA; 5 University of Washington School of Medicine, Seattle, WA USA

**Keywords:** Anxiety, Depression, Health Perception, Cardiovascular Disease, Stroke

## Abstract

**Background:**

While understanding the impact of mental health on health perception improves patient-centered care, this relationship is not well-established in patients with cardiovascular disease (CVD). We examined the relationship between psychological distress and health perception in patients with a previous myocardial infarction (MI) and/or stroke.

**Methods:**

We extracted data for patients with a previous MI and/or stroke from the 2019 National Health Interview Survey (NHIS). Health perception was self-reported. Presence and severity of anxiety and depression were estimated using the Generalized Anxiety Disorder-7 (GAD-7) and Patient Health Questionnaire-8 (PHQ-8). Binary analyses of anxiety/depression, multivariable logistic regressions controlling for confounders, and univariable analyses of confounders and anxiety/depression severity were performed.

**Results:**

Of 31,948 individuals for whom data on MI/stroke was available, 1235 reported a previous MI and 1203 a previous stroke. The odds of positive perceived health status were lower for individuals with anxiety/depression compared to those without anxiety/depression in both post-MI (anxiety OR 0.52, 95% CI = 0.32–0.85, P < 0.001; depression OR 0.45, 95% CI = 0.29–0.7, P < 0.001) and post-stroke groups (anxiety OR 0.61, 95% CI = 0.39–0.97, P < 0.001; depression OR 0.37, 95% CI = 0.25–0.55, P < 0.001) upon multivariable analyses. Increasing severity of anxiety/depression was also associated with worse perception of health status upon univariable analysis.

**Conclusion:**

Among patients with a previous acute CVD event, those with psychological distress have worse perception of their health status. Understanding the range of patient health perceptions can help physicians provide more patient-centered care and encourage patient behaviors that may improve both CVD and mental health outcomes.

## Background

Cardiovascular diseases (CVD) are leading causes of mortality in the US, and the relationship between CVD events, defined as myocardial infarction (MI) and stroke, with anxiety and depression is at the forefront of discussions about mental health in cardiovascular medicine [1–3]. Surviving an MI or stroke can lead to a long road to recovery for patients, which may be even more arduous when a survivor suffers from anxiety or depression [7, 8]. This is especially relevant given increased rates of anxiety and depression and increased incidence of coronary artery disease observed with the COVID-19 pandemic [9–11].

However, little is known about the factors related to how patients represent their own illness experiences in CVD event survivors [4–6]. According to Leventhal’s common sense model, illness perceptions can influence how patients cope with illness – a patient’s level of self-esteem and agency to perform protective health behaviors could directly affect outcomes [12]. For example, a systematic review found increased attendance to cardiac rehabilitation in MI survivors who view their illness as controllable [13].

Little is known on the relationship between illness perception and anxiety or depression in CVD event survivors, so we used nationally representative data from the National Health Interview Survey (NHIS) to highlight factors related to health perception in patients with a previous CVD event. We examined the relationship between the presence and severity of anxiety and depression with health perception in these populations.

## Methods

### Data source and sample

We analyzed data from the 2019 NHIS Sample Adult Interview, a nationally representative dataset of US adults [14]. The survey is published annually by the US Center for Disease Control and Prevention; methods have previously been published [14]. In short, the cross-sectional household interview survey of non-institutionalized US civilians is conducted annually in a face-to-face format.

In 2019, the survey was redesigned to collect data on self-health perception, anxiety, and depression using validated screening tools: the Generalized Anxiety Disorder-7 (GAD-7) and Patient Health Questionnaire-8 (PHQ-8). These were in addition to the continued collection of demographic data [15–17].

In 2019, 31,997 adults completed the survey, with a response rate of 59.1%. We included participants with a response of “yes” to having ever been told by a healthcare professional that they had an MI/stroke (n = 2,436).

### Measures

#### Health perception and psychological distress

Health perception was assessed by the question: “Would you say your health in general is excellent, very good, good, fair, or poor?” Anxiety and depression presence and severity were estimated using GAD-7 and PHQ-8 according to NHIS severity cutoffs: none/minimal (values 0–4), mild (values 5–9), moderate (values 10–14), and severe (values > 14) [15–17]. Presence of anxiety and depression were defined by a GAD-7 or PHQ-8 score greater than 4, respectively.

#### Statistical analyses

Subjects without a response for health perception, anxiety, depression, MI, or stroke were excluded. Anxiety and depression prevalence were reported as a percentage of respondents. Logistic regression analyses were conducted to determine the relationships between anxiety or depression and negative or positively perceived health status (NPHS or PPHS) defined as having answered “good”, “very good”, or “excellent” to the health perception survey question. Univariable analyses evaluated the association of anxiety/depression severity and other variables, including age, sex, ethnicity, BMI, household income, insurance status, and number of other comorbidities, with PPHS, separately for patients with prior MI and stroke. Then, multivariable models of the relationship between depression/anxiety and PPHS were constructed that adjusted for age, sex, race, ethnicity, BMI, household income, insurance status, and number of comorbidities.

All analyses were performed using Stata BE [18]. NHIS sampling weights were applied to provide nationally representative estimates and standard errors accounting for the complex sample design. We denote statistical significance with the following symbol: “*”.

## Results

### Sample characteristics

Of those with a previous MI (n = 1235, sample-weighted estimate, 7,864,416), approximately 48.1% reported having PPHS. Of those with a previous stroke (n = 1203, sample-weighted estimate, 7,741,516), 44.8% reported PPHS. Tables 1 and 2 show the characteristics of respondents.

**Table 1:** Characteristics of Patients with Previous Myocardial Infarction, with NHIS-Weighted Population Estimates

Characteristic	Negative Perceived Health Status		Positive Perceived Health Status		P-value
	Sample Data n = 617	Population estimate (%) N = 4,079,324	Sample Data n = 618	Population estimate (%) N = 3,785,093	
Age, y			(n = 617)	(N = 3,781,769)	0.07
Less than 65	236	1,691,970 (41.5%)	183	1,368,454 (36.2%)	
65–74	194	1,234,047 (30.2%)	181	1,069,524 (28.3%)	
75 or greater	187	1,153,307 (28.3%)	253	1,343,790 (35.5%)	
Sex					
Male	365	2,456,363 (60.4%)	407	2,544,775 (67.2%)	0.037*
Female	252	1,613,961 (39.6%)	211	1,240,318 (32.8%)	
Ethnicity					
NH White	445	2,932,752 (71.9%)	528	3,189,951 (84.3%)	
NH Black/AA	77	533,069 (13.1%)	42	277,632 (7.3%)	
Hispanic	60	422,846 (10.4%)	27	169,076 (4.5%)	0.000*
NH Asian	8	37,294 (0.9%)	10	96,192 (2.5%)	
NH AIAN	12	82,848 (2.0%)	3	15,103 (0.4%)	
Other or Multiple	15	70,514 (1.7%)	8	37,139 (1.0%)	
BMI Category	(n = 602)	(N = 3,988,243)	(n = 608)	(N = 3,741,539)	
Underweight	4	28,440 (0.7%)	7	40,588 (1.1%)	
Healthy Weight	149	948,935 (23.8%)	153	851,248 (22.8%)	< 0.001
Overweight	180	1,217,137 (30.5%)	259	1,631,042 (43.6%)	
Obese	269	1,793,730 (45.0%)	189	1,218,662 (32.6%)	
Household Income					
Less than 40,000	420	2,549,439 (62.5%)	250	1,330,863 (36.2%)	
40,000–79,999	137	1,022,048 (25.1%)	206	1,366,359 (36.1%)	< 0.001
80,000 or greater	60	507,837 (12.4%)	162	1,087,871 (28.7%)	
Insurance Status	(n = 615)	(N = 4,067,637)	(n = 618)		
Government Subsidized	303	1,976,487 (48.6%)	231	1,338,959 (35.4%)	
Private	198	1,333,765 (32.8%)	308	1,975,313 (52.2%)	< 0.001
Uninsured	13	112,167 (2.8%)	16	113,826 (3.0%)	
Other	101	645,219 (15.9%)	63	356,994 (9.4%)	
No. of Other Comorbidities	(n = 610)	(N = 4,034,763)	(n = 616)	(N = 3,770,986)	
Zero	11	49,545 (1.2%)	29	202,181 (5.4%)	
One	48	332,947 (8.3%)	85	518,959 (13.8%)	
Two	95	555,860 (13.8%)	194	1,203,247 (31.9%)	< 0.001
Three	171	1,223,787 (30.3%)	178	1,069,762 (28.4%)	
Four	168	1,070,690 (26.5%)	101	622,103 (16.5%)	
Five	89	568,820 (14.1%)	26	130,893 (3.5%)	
Six	28	233,114 (5.8%)	3	23,840 (0.6%)	
Anxious	(n = 589)	(N = 3,895,450)	(n = 606)	(N = 3,695,611)	< 0.001
None/Minimal	396	2,540,959 (65.2%)	549	3,287,258 (89.0%)	
Mild	90	591,295 (15.2%)	39	272,031 (7.4%)	
Moderate	46	396,893 (10.2%)	10	74,921 (2.0%)	
Severe	57	366,303 (9.4%)	8	61,401 (1.7%)	
Depressed	(n = 594)	(N = 3,932,091)	(n = 606)	(N = 3,695,611)	< 0.001
None/Minimal	297	1,995,944 (50.8%)	510	3,042,245 (82.3%)	
Mild	149	918,198 (23.4%)	67	438,519 (11.9%)	
Moderate	77	527,900 (13.4%)	19	149,842 (4.1%)	
Severe	71	490,049 (12.5%)	10	65,005 (1.8%)	

* NH = “non-Hispanic”; AIAN = “American Indian/Alaska Native”

**Table 2:** Characteristics of Patients with Previous Stroke, with NHIS-Weighted Population Estimates

Characteristic	Negative Perceived Health Status		Positive Perceived Health Status		P-value
	Sample Data n = 622	Population estimate (%) N = 4,271,824	Sample Data n = 581	Population estimate (%) N = 3,469,692	
Age, y	(n = 621)	(N = 4,269,701)	(n = 579)	(N = 3,449,436)	0.05
Less than 65	246	2,016,008 (47.2%)	183	1,349,061 (39.1%)	
65–74	166	989,993 (23.2%)	157	927,058 (26.9%)	
75 or greater	209	1,263,701 (29.6%)	239	1,173,317 (34.0%)	
Sex					
Male	290	2,104,265 (49.3%)	263	1,586,891 (45.7%)	0.36
Female	332	2,167,559 (50.7%)	318	1,882,802 (54.3%)	
Ethnicity					
NH White	414	2,651,356 (62.1%)	444	2,539,285 (73.2%)	
NH Black/AA	122	869,480 (20.4%)	81	554,001 (16.0%)	
Hispanic	56	559,959 (13.1%)	33	230,447 (6.6%)	0.009*
NH Asian	8	50,477 (1.2%)	12	74,011 (2.1%)	
NH AIAN	8	43,147 (1.0%)	4	21,483 (0.6%)	
Other or Multiple	14	97,405 (2.3%)	7	50,465 (1.5%)	
BMI Category	(n = 611)	(N = 4,210,402)	(n = 572)	(N = 3,403,960)	
Underweight	11	81,669 (1.9%)	9	46,697 (1.4%)	
Healthy Weight	175	1,137,001 (27.0%)	140	729,211 (21.4%)	0.12
Overweight	187	1,202,752 (28.6%)	209	1,189,670 (35.0%)	
Obese	238	1,788,980 (42.5%)	214	1,438,382 (42.3%)	
Household Income					
Less than 40,000	433	2,715,477 (63.6%)	299	1,557,471 (44.9%)	
40,000–79,999	141	1,137,010 (26.6%)	167	1,064,655 (30.7%)	< 0.001
80,000 or greater	48	419,337 (9.8%)	115	847,567 (24.4%)	
Insurance Status	(n = 620)	(N = 4,267,246)	(n = 580)	(N = 3,461,985)	
Government Subsidized	326	2,183,765 (51.2%)	232	1,262,767 (36.5%)	
Private	186	1,286,911 (30.2%)	243	1,523,594 (44.0%)	< 0.001
Uninsured	23	188,587 (4.4%)	26	184,777 (5.3%)	
Other	85	607,983 (14.3%)	79	490,846 (14.2%)	
No. of Other Comorbidities	(n = 610)	(N = 4,196,417)	(n = 575)	(N = 3,421,550)	
Zero	26	195,078 (4.7%)	87	574,704 (16.8%)	
One	75	494,879 (11.8%)	135	715,073 (20.9%)	
Two	166	1,243,296 (29.6%)	174	1,029,174 (30.1%)	< 0.001
Three	158	1,042,697 (24.9%)	122	737,775 (21.6%)	
Four	128	834,033 (19.9%)	41	261,718 (7.7%)	
Five	43	262,379 6.3%)	16	103,106 (3.0%)	
Six	14	124,054 (3.0%)	0	0 (0.0%)	
Anxious	(n = 589)	(N = 4,016,247)	(n = 568)	(N = 3,370,440)	< 0.001
None/Minimal	365	2,383,413 (59.3%)	492	2,870,285 (85.2%)	
Mild	101	710,577 (17.7%)	51	330,867 (9.8%)	
Moderate	54	399,893 (10.0%)	13	83,443 (2.5%)	
Severe	69	522,365 (13.0%)	12	85,846 (2.6%)	
Depressed	(n = 594)	(N = 4,061,905)	(n = 568)	(N = 3,370,440)	< 0.001
None/Minimal	274	1,803,702 (44.4%)	431	2,627,381 (78.0%)	
Mild	156	992,194 (24.4%)	90	460,823 (13.6%)	
Moderate	84	652,005 (16.1%)	30	170,478 (5.1%)	
Severe	80	614,004 (15.1%)	17	111,758 (3.3%)	

* NH = “non-Hispanic”; AIAN = “American Indian/Alaska Native”

#### Health perception in MI-survivors

Of the 1195 (sample weighted estimate = 7,591,061) post-MI adults with GAD-7 responses, 23.2% reported anxiety and of the 1200 (sample weighted estimate = 7,627,702) with PHQ-8 responses, 33.9% reported depression (Table 1). Univariable logistic regression analyses, with reference groups of none/minimal anxiety or depression, showed that mild [OR 0.36 (0.21, 0.59), p < 0.001*], moderate [OR 0.15 (0.06, 0.34), p < 0.001*], and severe anxiety [OR 0.13 (0.06,0.13), p < 0.001*], and mild [OR 0.31 (0.21, 0.47), p < 0.001*], moderate [OR 0.19 (0.10, 0.36), p < 0.001*], and severe depression [OR 0.09 (0.04, 0.18), p < 0.001*] were associated with NPHS (Table 3). A multivariable model confirmed that the presence of either anxiety or depression were associated with decreased odds of PPHS, but there were mixed results related to anxiety/depression severity (Table 4). In our multivariable models, the odds of self-reporting PPHS was 48% lower among those with anxiety [OR 0.52 (0.32, 0.85), p < 0.009*], and 55% lower among those with depression [OR 0.45 (0.29, 0.70), p < 0.001*] compared to those who did not report a mental health comorbidity (Table 4).

**Table 3:** Univariable Logistic Regression Results for Factors Related to Positive Perceived Health Status in Post-MI Patients

Characteristic	Odds Ratio (95% CI)	*P*-Value	
Age, y			
Less than 65 (n = 419)	Reference	-	
65–74 (n = 375)	1.07 (0.76, 1.51)	0.69	
75 or greater (n = 440)	1.44 (1.04, 2.00)	0.029*	
Sex			
Male (n = 772)	Reference	-	
Female (n = 463)	0.74 (0.56, 0.98)	0.037*	
Ethnicity			
NH White (n = 973)	Reference	-	
NH Black/AA (n = 119)	0.48 (0.30, 0.76)	0.002*	
Hispanic (n = 87)	0.37 (0.22, 0.61)	< 0.001*	
NH Asian (n = 18)	2.37 (0.87, 6.43)	0.09	
NH AIAN (n = 15)	0.17 (0.04, 0.68)	0.012*	
Other or Multiple (n = 23)	0.48 (0.17, 1.35)	0.17	
BMI Category			
Underweight (n = 11)	1.59 (0.38, 6.73)	0.53	
Healthy Weight (n = 302)	Reference	-	
Overweight (n = 439)	1.49 (1.03, 2.17)	0.034*	
Obese (n = 458)	0.76 (0.54, 1.06)	0.10	
Household Income			
Less than 40,000 (n = 670)	Reference	-	
40,000–79,999 (n = 343)	2.56 (1.87, 3.51)	< 0.001*	
80,000 or greater (n = 222)	4.10 (2.71, 6.22)	< 0.001*	
Insurance Status			
Government Subsidized (n = 534)	Reference	-	
Private (n = 506)	2.19 (1.63, 2.94)	< 0.001*	
Uninsured (n = 29)	1.50 (0.64, 3.50)	0.35	
Other (n = 164)	0.82 (0.52, 1.28)	0.38	
No. of Other Comorbidities			
Zero (n = 40)	Reference	-	
One (n = 133)	0.38 (0.15, 1.00)	0.05	
Two (n = 289)	0.53 (0.21, 1.31)	0.17	
Three (n = 349)	0.21 (0.09, 0.51)	0.001*	
Four (n = 269)	0.14 (0.06, 0.35)	< 0.001*	
Five (n = 115)	0.06 (0.02, 0.15)	< 0.001*	
Six (n = 31)	0.03 (0.01, 0.12)	< 0.001*	
Anxious			
None/Minimal (n = 945)	Reference	-	
Mild (n = 129)	0.36 (0.21, 0.59)	< 0.001*	
Moderate (n = 56)	0.15 (0.06, 0.34)	< 0.001*	
Severe (n = 65)	0.13 (0.06, 0.29)	< 0.001*	
Depressed			
None/Minimal (n = 807)	Reference	-	
Mild (n = 216)	0.31 (0.21, 0.47)	< 0.001*	
Moderate (n = 96)	0.19 (0.10, 0.36)	< 0.001*	
Severe (n = 81)	0.09 (0.04, 0.18)	< 0.001*	

**Table 4:** Adjusted Multivariable Logistic Regression of Anxiety/Depression with Positive Perceived Health Status in Previous MI and Previous Stroke Groups

Characteristic	Odds Ratio	P-value
Previous MI (n = 1235)		
Anxious		
None/Minimal	Reference	-
Mild	0.65 (0.37,1.15)	0.136
Moderate	0.31 (0.10,1.01)	0.052
Severe	0.59 (0.20,1.76)	0.347
Mild, Moderate, or Severe	0.52 (0.32, 0.85) *	0.009
Depressed		
None/Minimal	Reference	-
Mild	0.51 (0.31,0.83)	0.007
Moderate	0.50 (0.22,1.11)	0.222
Severe	0.30 (0.91,2.09)	0.120
Mild, Moderate, or Severe	0.45 (0.29, 0.70) *	< 0.001
Previous Stroke (n = 1203)		
Anxious		
None/Minimal	Reference	-
Mild	0.77 (0.45, 1.30)	0.324
Moderate	0.56 (0.25,1.27)	0.165
Severe	0.36 (0.14,0.91)	0.031
Mild, Moderate, or Severe	0.61 (0.39, 0.97) *	0.037
Depressed		
None/Minimal	Reference	-
Mild	0.39 (0.25,0.61)	< 0.001
Moderate	0.34 (0.18,0.67)	0.002
Severe	0.41 (0.18,0.92)	0.031
Mild, Moderate, or Severe	0.37 (0.25, 0.55) *	< 0.001

*(Adjusted for age, sex, race/ethnicity, BMI, Household income, Insurance status, and Number of comorbidities, Anxiety, and Depression)

### Post-stroke health perception

Of the 1157 (sample weighted estimate = 7,386,687) post-stroke patients with GAD-7 responses, 28.9% reported anxiety, and of the 1162 (sample weighted estimate = 7,432,345) with PHQ-8 responses, 40.4% reported depression. Similarly to post-MI patients, those with mild [OR 0.39 (0.23, 0.63), p < 0.001*], moderate [OR 0.17 (0.08, 0.35), p < 0.001*], and severe [OR 0.14 (0.07, 0.28), p < 0.001*] anxiety, and mild [OR 0.32 (0.22, 0.46), p < 0.001*], moderate [OR 0.18 (0.10, 0.32), p < 0.001*], and severe [OR 0.12 (0.06, 0.24), p < 0.001*] depression demonstrated a negative association between anxiety/depression and PPHS compared to subjects with none/minimal anxiety or depression (Table 5). Multivariable analysis revealed that the odds of reporting PPHS was 39% lower among those with anxiety [OR 0.61 (0.39, 0.97), p < 0.037*] and 63% lower among those with depression [OR 0.37 (0.25, 0.55), p < 0.001*] compared those without anxiety or depression, respectively; there were mixed results related to the severity of anxiety and depression (Table 4).

**Table 5:** Univariate Logistic Regression Results for Factors Related to Positive Perceived Health Status in Post-Stroke Patients

Characteristic	Odds Ratio (95% CI	*P*-Value
Age, y		
Less than 65 (n = 429)	Reference	-
65–74 (n = 323)	1.40 (0.98, 2.00)	0.69
75 or greater (n = 448)	1.39 (1.02, 1.88)	0.035*
Sex		
Male(n = 553)	Reference	-
Female (n = 650)	1.15 (0.85, 1.56)	0.36
Ethnicity		
NH White (n = 858)	Reference	-
NH Black/AA (n = 203)	0.67 (0.67, 0.97)	0.034*
Hispanic (n = 89)	0.43 (0.24, 0.77)	0.004*
NH Asian (n = 20)	1.53 (0.48, 4.91)	0.47
NH AIAN (n = 12)	0.52 (0.13, 2.08)	0.35
Other or Multiple (n = 21)	0.54 (0.18, 1.59)	0.26
BMI Category		
Underweight (n = 20)	0.89 (0.31, 2.59)	0.83
Healthy Weight (n = 315)	Reference	-
Overweight (n = 396)	1.54 (1.00, 2.18)	0.014*
Obese (n = 452)	1.25 (0.88, 1.79)	0.22
Household Income		
Less than 40,000 (n = 315)	Reference	-
40,000–79,999 (n = 308)	1.63 (1.19, 2.24)	0.002*
80,000 or greater (n = 163)	3.52 (2.18, 5.69)	< 0.001*
Insurance Status		
Government Subsidized (n = 558)	Reference	-
Private (n = 429)	2.05 (1.51, 2.78)	< 0.001*
Uninsured (n = 49)	1.69 (0.81, 3.53)	0.16
Other (n = 164)	1.40 (0.80, 2.19)	0.15
No. of Other Comorbidities		
Zero (n = 113)	Reference	-
One (n = 210)	0.49 (0.26, 0.93)	0.030*
Two (n = 340)	0.28 (0.15, 0.53)	< 0.001*
Three (n = 278)	0.24 (0.13, 0.44)	< 0.001*
Four (n = 169)	0.11 (0.05, 0.22)	< 0.001*
Five (n = 59)	0.13 (0.05, 0.33)	< 0.001*
Six (n = 14)	Null (predicted negative perfectly)	-
Anxious		
None/Minimal (n = 857)	Reference	-
Mild (n = 152)	0.39 (0.23, 0.63)	< 0.001*
Moderate (n = 67)	0.17 (0.08, 0.35)	< 0.001*
Severe (n = 81)	0.14 (0.07, 0.28)	< 0.001*
Depressed		
None/Minimal (n = 705)	Reference	-
Mild (n = 246)	0.32 (0.22, 0.46)	< 0.001*
Moderate (n = 114)	0.18 (0.10, 0.32)	< 0.001*
Severe (n = 97)	0.12 (0.06, 0.24)	< 0.001*

### Other factors related to health perception

Minority groups were more likely to report NPHS in a univariable analysis. Among self-identified Non-Hispanic (NH) Black/African American (AA) individuals, the odds of reporting PPHS was 52% lower among post-MI participants [OR 0.48 (0.30, 0.76), p < 0.002*] and 33% lower among post-stroke participants [OR 0.67 (0.67, 0.97, p < 0.034*] compared to self-identified NH White individuals (Tables 3 and 5). Tables 3 and 5 further describe factors that may be related to health status perception in univariable analyses.

## Discussion

Our analysis demonstrates that anxiety and depression are significantly associated with NPHS in a large, nationally representative sample of US adults with a previous MI/stroke. Additionally, subjects with higher severity of anxiety and depression may be more likely to have NPHS in our sample. By demonstrating the relationship between mental illness and illness perception in survivors of different CVD events, we hope that this study encourages providers to address health perception and mental illness, and informs the development of interventions to improve mental health and behavior change in CVD event survivors.

### Relationship between CVD events and psychological distress

While Leventhal’s common sense theory suggests the limitation of patient behavior change and action in accordance with their treatment plan when overcome by fear or lack of the feeling that their actions can change their outcomes, the evidence on interventions to affect health perception and behavior change, and factors that may be related to health perception is currently limited [12].

We know that psychological distress in post-MI patients has been associated with poorer health outcomes and higher rates of anginal symptoms at one year post-MI [19]. One additionally found that self-perception of high stress in a patient’s life correlates with high levels of psychological distress and increases the risk of premature death [20].

Prior to our study, it was unknown whether psychological distress and health perception in MI survivors were related. In our study, those with NPHS were more likely to have anxiety/depression: 65.2% of post-MI patients with NPHS reported none/minimal anxiety, while 89% of post-MI patients with PPHS reporting none/minimal anxiety. Similarly, 50.8% of post-MI patients with NPHS reported none/minimal depression, compared to 82.3% of post-MI patients with PPHS and none/minimal depression.

It is also well known that depressed mood can affect stroke survivors [21, 22]. Poor social support and psychological distress increase depressive symptoms in post-stroke patients [23]. Anxiety is also common following a stroke, especially in the first year [24]. Among post-stroke patients in our study, 59.3% with NPHS and 85.2% with PPHS reported none/minimal anxiety, while 44.4% of those with NPHS and 78% with PPHS who reported none/minimal depression. Additionally, in this study, increased severity of anxiety/depression correlated with worse perceived health status in post-MI and post-stroke groups.

Acknowledging that a relationship exists between mental health and illness perception can inform the design of future interventions to improve post-MI and post-stroke care. While we are unable to assess temporality due to the cross-sectional design of this study, the information we provide supports a holistic approach to addressing both illness perception and mental health; addressing one may lead to a favorable response in the other. Encouraging results from a previous randomized control trial of an illness perception intervention showed that better understanding of their condition, more favorable illness perception, and earlier return to work in the group that received the intervention [25].

*Relationship between other variables and health perception*Others who may be at risk for worse health perception in our study include those of age < 75, some minority races/ethnicity groups, higher BMI, lower income, and those with an increasing number of comorbidities. Age over 75 was associated with higher odds of reporting PPHS. This differs from previous findings, where older age was associated with a decline in subjective health ratings [26, 27]. However, those studies found several moderators of these associations, including cognitive functioning, presence of physical illness, and religiosity [26, 28, 29].

Post-MI and post-stroke patients identifying as NH Black/AA or Hispanic had significantly lower odds of reporting PPHS than NH White patients (Tables 1 and 2). One cross-sectional study of US adults also found that NH Black/AA patients had a higher prevalence of poor/fair perceived health status compared to NH White patients [30]. Our findings may provide insight into health status perception as an additional potential mediator of racial/ethnic disparities in adverse cardiovascular events. One large observational cohort study found that Black patients had 72% higher CVD mortality hazard after adjusting for age and sex than White patients [33]. Further investigation should be conducted to better understand the perception of health status in Black, Hispanic, and other minority populations, and how to address health perception in addition to the social determinants of health to minimize the existing disparities in adverse cardiovascular outcomes.

Finally, household income was found to be related to PPHS in both groups, while being overweight was associated with NPHS in post-MI patients (Tables 1 and 2). This supports previous research showing individuals with higher BMI are less likely to view themselves as healthy [31, 32]. One study found that self-perceived income had a greater impact on health perception than objective income level [34].

Our findings reinforce the need for mental health screening and treatment in patients who experience an adverse CVD event. Perception of health status can influence health behaviors, the processing of health information, and healthcare decision making [35]. This includes the adherence to cardiac rehabilitation and post-MI or post-stroke care, which are crucial to achieving positive cardiovascular outcomes for patients [13]. By addressing patients’ mental health and promoting more positive health status perception, providers can improve the cardiovascular and psychological outcomes of their patients after a CVD event. Thus, it is important that providers address health perception in addition to anxiety and depression in CVD event survivors.

### Limitations

The limitations of this study include the limitations of the NHIS 2019, as previously described [14, 15]. These include selection and non-response bias, as well as that data is self-reported. We applied sample weights provided by the NHIS to attenuate the risk of non-response bias. We use responses to the NHIS “rotating core” of questions not asked annually, therefore 2019 data was the most recently available data at the time this analysis was conducted. The relationships observed in this study may have been modified by the COVID-19 pandemic. We dichotomized variables to form the variables “anxious”, “depressed”, “PPHS”, and “NPHS”, to perform logistic regression analyses, thus we do not account for different levels of health perception. The cross-sectional nature of this analysis is an additional limitation, as we cannot assess temporality to understand if psychological distress led to illness perception or vice versa. Finally, we do not have longitudinal follow-up on subsequent health outcomes, so we cannot assess to what degree health perception mediates the known relationship between mood disorders and outcomes in patients who have had an MI or stroke.

## Conclusions

Given the high prevalence of anxiety and depression among MI and stroke survivors, it is essential that providers consider the impact of mental health and illness perceptions on patient outcomes. Through a nationally representative dataset, we suggest that anxiety and depression are associated with worse illness perception in CVD event survivors, and that race, ethnicity, age, and other factors are also related to health status perception. Given the influence of illness perception on health behaviors and treatment plan adherence, it is crucial that providers address the mental health and health perception of their CVD patients.

## Data Availability

The datasets generated and/or analyzed during the current study are available in the NHIS repository, https://www.cdc.gov/nchs/nhis/2019nhis.htm.

## Abbreviations

CVD: Cardiovascular disease
MI: Myocardial infarction
NHIS: National Health Interview Survey
GAD-7: Generalized Anxiety Disorder-7
PHQ-8: Patient Health Questionnaire-8
NPHS: Negative perceived health status
PPHS: Positively perceived health status
NH: Non-Hispanic
AA: African American
AIAN: American Indian/Alaska Native
CDC: Centers for Disease Control and Prevention
